# Beyond hypoxic–ischemic encephalopathy: genetic insights and precision diagnosis in neonatal encephalopathies

**DOI:** 10.3389/fmed.2026.1868036

**Published:** 2026-06-15

**Authors:** Carla Cimino, Vincenzo Sortino, Annamaria Sapuppo, Marco Andrea Nicola Saporito, Federica Scarlata, Raffaele Falsaperla

**Affiliations:** 1 Unit of Neonatal Intensive Care and Neonatology, University Hospital Policlinico “G.Rodolico-San Marco, ” Catania, Italy; 2 National Council of Research Institute for Research, Institute for Research and Biomedical Innovation (IRIB), Unit of Catania, Catania, Italy; 3 PhD Program in Innovative Technologies in Biomedical Sciences, University Kore of Enna, Enna, Italy; 4 Unit of Pediatrics and Pediatric Emergency, Azienda Ospedaliero-Universitaria Policlinico “G.Rodolico-San Marco”, San Marco Hospital, University of Catania, Catania, Italy; 5 Postgraduate Training Programme in Pediatrics, Department of Clinical and Experimental Medicine, University of Catania, Catania, Italy; 6 Department of Medical Science-Pediatrics, University of Ferrara, Ferrara, Italy

**Keywords:** genetic etiology, hypoxic–ischemic encephalopathy, neonatal encephalopathy, next-generation sequencing, precision medicine

## Abstract

Neonatal encephalopathies are a heterogeneous group of early-onset neurological disorders. While hypoxic-ischemic encephalopathy (HIE) has long been considered the predominant cause, a growing number of genetic, metabolic, immune-mediated, and toxic etiologies may clinically mimic HIE, posing diagnostic challenges. Timely recognition of these alternative causes is essential to initiate targeted therapies and provide appropriate family counseling. This article reviews the main clinical presentations and pathogenetic mechanisms of neonatal encephalopathies and introduces a structured diagnostic algorithm, presented as a flow chart, to guide neonatologists in early differential diagnosis and precision management of affected newborns.

## Introduction

Neonatal encephalopathy (NE) is defined as a heterogeneous neurological syndrome occurring in the first days of life, characterized by alteration in mental status and/or seizures, often associated with hypotonia, generalized weakness, decreased axial and/or appendicular tone, and respiratory distress, with or without multisystem involvement (1).

This definition reflects the multifactorial and clinically variable nature of NE and highlights the need for a broad and in-depth diagnostic approach beyond the hypoxic–ischemic hypothesis (2). Historically, NE has been predominantly conceptualized within the framework HIE, resulting in a clinical approach largely centered on perinatal evaluation and the implementation of therapeutic hypothermia in moderate to severe cases (3).

However, recent evidence shows a more complex reality. A growing number of encephalopathic neonates do not present clinical, biochemical, or neuroradiological findings consistent with hypoxic–ischemic injury. In many cases, the etiology lies in a genetic basis, involving pathogenic variants affecting central nervous system development, neuronal excitability, synaptogenesis, ion channels, or mitochondrial metabolism (4). A similar diagnostic challenge may arise in cases of sudden unexpected postnatal collapse or acute postnatal deterioration, where hypoxic–ischemic injury and alternative metabolic or genetic etiologies may coexist or be difficult to be distinguished.

Genetic neonatal encephalopathies are not only an emerging category but also a growing clinical reality and diagnostic challenge (5). Some forms are potentially treatable in the neonatal period, such as vitamin-responsive encephalopathies, urea cycle disorders, and certain epileptic channelopathies (6). Early identification enables targeted therapy, avoids inappropriate treatments, and improves genetic counseling. Additionally, genetic variants may act as susceptibility factors to hypoxic–ischemic injury (7). In these cases, even mild insults can produce disproportionately severe brain damage due to underlying molecular vulnerabilities.

Next-generation sequencing (NGS), including targeted panels and whole-exome sequencing (WES), and, increasingly, whole-genome sequencing (WGS), is now a first-line diagnostic tool in neonatal encephalopathy (8). This approach should be integrated with EEG/aEEG, brain Magnetic Resonance Imaging (MRI), and metabolic evaluation to achieve a multidimensional diagnostic framework. The aim of this review is to provide an updated guide for pediatricians and neonatologists to recognize, classify, and treat genetic neonatal encephalopathies. It reviews the main early-onset forms, explores genes associated with susceptibility to HIE, and discusses the clinical and therapeutic implications of early molecular diagnosis.

The literature review was conducted searching on PubMed, Scopus, and Embase keywords related to NE, genetics, HIE, metabolic and mitochondrial disorders, and advanced genomic techniques. Studies published between 2012 and 2025 were included, with priority given to original research, systematic reviews, and clinically relevant contributions. A total of 214 records were identified, of which 176 were screened after removal of duplicates. Following title and abstract screening, 35 articles were included based on relevance to the scope of this review. Of interest, advances in clinical genomics have led to the identification of genetic neonatal encephalopathies as a distinct group. These conditions comprise a heterogeneous set of monogenic disorders, caused by pathogenic variants in a single gene, with onset in the first days of life, characterized by acute neurological dysfunction. They may either mimic HIE or represent distinct clinical entities with specific pathophysiological mechanisms. A functional classification into five major groups is proposed, based on clinical and diagnostic features. (Table 1)

**TABLE 1:** Clinical and genetic classification of neonatal encephalopathies: key features for differential diagnosis.

Clinical–genetic category	Genes involved (examples)	Main clinical features
Epileptic channelopathies	*KCNQ2, SCN2A, SCN8A, KCNT1*	Early-onset seizures, burst-suppression EEG, normal or nonspecific MRI
Genes of neuronal development and synaptogenesis	*STXBP1, CDKL5, ARX, TUBA1A, KIF1A, MECP2*	Hypotonia, early encephalopathy, central apnea, normal MRI or mild dysgenesis
Mitochondrial and metabolic dysfunctions	*POLG, MTFMT, SUCLA2, PDHA1*	Lactic acidosis, refractory seizures, metabolic decompensation
Treatable inborn errors of metabolism	*OTC, ASS1, GLDC, BTD, MOCS1*	Lethargy, seizures, hyperammonemia or metabolic abnormalities, treatable conditions
Vitamin-dependent and/or responsive encephalopathies	*ALDH7A1, PNPO, SLC19A3*	Drug resistant seizures, hypotonia, response to vitamin B6 or cofactor

## Classification of genetic neonatal encephalopathies

A classification includes five main groups:

1. Encephalopathies due to genes affecting neuronal excitability and ion channels
2. Encephalopathies due to genes involved in neuronal development and synaptogenesis
3. Encephalopathies due to genes affecting mitochondrial and energy metabolism
4. Encephalopathies due to treatable inborn errors of metabolism
5. Encephalopathies due to genetic conditions associated with HIE phenocopies or increased susceptibility to hypoxic injury

## Encephalopathies due to genes affecting neuronal excitability and ion channels

Channelopathies are among the most relevant genetic causes of neonatal encephalopathy and represent the leading monogenic cause of neonatal epileptic encephalopathy, accounting for up to 25–30% of early drug-resistant seizures (9). The *KCNQ2* gene is most frequently involved and encodes a potassium channel critical for neuronal stability. Clinically, *KCNQ2*-related encephalopathies present with tonic seizures with onset within the first 24–72 h of life, often associated with a burst-suppression EEG pattern and severe hypotonia (10). Pathogenic variants in *SCN2A*, encoding a sodium channel, cause seizures within the first days of life and may respond to sodium channel blockers such as carbamazepine (11). More severe phenotypes are associated with *SCN8A*, often presenting later in the neonatal period and showing limited therapeutic response (12). Finally, *KCNT1* pathogenic variants lead to severe, drug-resistant epilepsy with early onset and poor prognosis (13). The main neonatal epileptic channelopathies, including their clinical features, age at onset, and response to targeted therapies, are summarized in Table 2.

**TABLE 2:** Main channelopathies associated with neonatal encephalopathy/genes affecting neuronal excitability and ion channels.

Gene	Ion channel type	Typical age at onset	Main clinical features	Targeted pharmacological response	Relative frequency	Initial symptoms
*KCNQ2*	Potassium channel (M-type)	24–72 hours	Tonic seizures, burst-suppression, hypotonia	Retigabine, carbamazepine	High (most frequent)	Generalized tonic seizures, apnea, hypotonia
*SCN2A*	Sodium channel (NaV1.2)	1–2 days	Multifocal seizures, burst-suppression	Carbamazepine, oxcarbazepine	Moderate	Clonic or multifocal seizures, irritability, poor feeding
*SCN8A*	Sodium channel (NaV1.6)	3–6 weeks	Tonic-clonic seizures, spasms, movement disorders	Phenytoin (variable response)	Low	Tonic-clonic seizures, inconsolable crying, tone abnormalities
*KCNT1*	Sodium-activated potassium channel	Weeks 1–4	Migrating focal seizures, severe drug resistance	Quinidine (off-label)	Rare	Multiple focal seizures, poor responsiveness, autonomic instability

## Encephalopathies due to genes involved in neuronal development and synaptogenesis

Pathogenic variants in genes regulating neuronal migration, cortical organization, and synaptic function cause early and severe encephalopathies (14).

- *STXBP1* pathogenic variants result in early epileptic encephalopathy with burst-suppression EEG patterns. Some patients show a partial response to levetiracetam or valproate, but the overall prognosis remains poor (15).
- *CDKL5* deficiency disorder presents with seizures within the first weeks of life and severe neurodevelopmental impairment. Loss-of-function pathogenic variants cause a condition known as *CDKL5* Deficiency Disorder (CDD), previously classified as an early-onset variant of Rett syndrome. The typical onset occurs within the first 6 weeks of life, with multifocal epileptic seizures that are difficult to control and associated with severe hypotonia, poor interaction, and subsequent neuromotor impairment (16).
- *ARX* pathogenic variants are associated with brain malformations and severe epilepsy. The clinical presentation is typically neonatal, characterized by severe hypotonia, drug-resistant epileptic seizures, and cerebral dysgenesis. Prognosis is, often, poor and no causal therapies are currently available, although some patients may show a partial response to vigabatrin or corticosteroids (17).
- *TUBA1A* pathogenic variants cause a broad spectrum of brain development disorders, including lissencephaly, pachygyria, corpus callosum hypoplasia, and brainstem dysplasia. Neonatal onset is characterized by severe hypotonia, early epileptic seizures, and respiratory difficulties (18).
- *KIF1A* pathogenic variants lead to severe neurodevelopmental delay and early-onset seizures. Pathogenic variants, *de novo*, cause a syndromic form of early-onset encephalopathy characterized by psychomotor delay, microcephaly, hypotonia, and early refractory seizures (4).
- *MECP2* affects epigenetic regulation and synaptogenesis, producing phenotypes that may mimic HIE (19, 20).

The main genetic disorders affecting neuronal development and synaptogenesis, along with their clinical features and therapeutic approaches, are summarized in Table 3.

**TABLE 3:** Genetic disorders of neuronal development and synaptogenesis in neonatal encephalopathy/genes involved in neuronal development and synaptogenesis.

Gene	Function	Age at onset	Clinical features	Targeted treatment
*STXBP1*	Synaptic vesicle release (SNARE complex)	First 48–72 hours	Seizures, burst-suppression EEG, hypotonia	Levetiracetam, valproate (partial response)
*CDKL5*	Dendritic spine regulation and synaptic plasticity	Within first 6 weeks	Multifocal seizures, severe hypotonia, motor dysfunction	Gene therapy under investigation, symptomatic management
*ARX*	Neuronal migration and basal ganglia development	Early neonatal	Drug-resistant seizures, brain dysgenesis, hypotonia	Vigabatrin, corticosteroids (variable response)
*TUBA1A*	Neuronal microtubule assembly	Neonatal/prenatal	Seizures, hypotonia, brain malformations	Symptomatic and multidisciplinary care
*KIF1A*	Axonal transport of vesicles and mitochondria	Early neonatal	Early seizures, microcephaly, severe developmental delay	Preclinical AAV (Adeno-Associated Virus)-based therapies
*MECP2*	Epigenetic regulator of synaptogenesis	Early neonatal	Severe hypotonia, apnea	Preclinical gene therapy research

## Encephalopathies due to genes affecting mitochondrial and energy metabolism

Mitochondrial encephalopathies are severe conditions caused by defects in oxidative phosphorylation or energy metabolism.

Pathogenic variants in *POLG* (encoding mitochondrial DNA polymerase gamma) are among the most common and present with seizures, hypotonia, and liver dysfunction. A large cohort study showed that over 30% of neonates with suspected metabolic encephalopathy had mitochondrial-related pathogenic variants (21).

Other important genes include:

- *MTFMT*, affecting mitochondrial protein synthesis (22)
- *PDHA1*, causing pyruvate metabolism defects and lactic acidosis (23)
- *SUCLA2*, associated with severe metabolic encephalopathy (24)

## Encephalopathies due to treatable inborn errors of metabolism

Some neonatal encephalopathies are potentially treatable, making early diagnosis crucial. Examples include:

- Non-ketotic hyperglycinemia (*GLDC*) (25)
- Molybdenum cofactor deficiency (*MOCS1/2*) (26)
- Thiamine transporter deficiency (*SLC19A3*) (26)
- Pyridoxine-dependent epilepsy (*ALDH7A1, PNPO, PLPBP*) (27)
- Urea cycle disorders (*OTC, ASS1, ARG1*) (28)

In these cases, early targeted therapy can significantly improve outcomes or prevent irreversible damage

Key treatable genetic and metabolic causes of neonatal encephalopathy and their targeted therapies are summarized in Table 4

**TABLE 4:** Main treatable metabolic disorders associated with neonatal encephalopathy/treatable metabolic and vitamin-responsive disorders.

Disorder	Clinical clues	Diagnostic clue	Targeted therapy
Pyridoxine-dependent epilepsy	Refractory neonatal seizures	EEG burst suppression	Pyridoxine
Urea cycle disorders	Encephalopathy + apnea	Hyperammonemia	Nitrogen scavengers/dialysis
NKH	Hiccups + hypotonia	Elevated CSF glycine	Sodium benzoate
Molybdenum cofactor deficiency	Early seizures	Sulfite positive urine	cPMP

## Encephalopathies due to genetic conditions associated with HIE phenocopies or increased susceptibility to hypoxic injury

Recent studies have shown that a significant proportion of neonates diagnosed with HIE actually have underlying genetic conditions. In a retrospective exome sequencing study, a genetic diagnosis was identified in 25% of cases initially classified as HIE (4).

These genes may either:

- mimic HIE phenotypically
- increase susceptibility to hypoxic injury

This has led to a paradigm shift toward a “two-hit model,” combining genetic vulnerability and environmental stress. For example, heterozygous pathogenic variants in *POLG* impair oxidative phosphorylation capacity, leaving neurons with a reduced bioenergetic reserve so that even a mild hypoxic insult can trigger disproportionate oxidative stress and cellular injury (29). Similarly, heterozygous pathogenic variants in *SCN1A*, encoding the NaV1.1 sodium channel subunit, lower seizure threshold via impaired inhibitory interneuron function, meaning a transient hypoxic episode insufficient to cause encephalopathy in a neurotypical neonate may precipitate refractory seizures and secondary neuronal damage in a genetically susceptible infant (30). Selected genetic conditions that may mimic hypoxic–ischemic encephalopathy, along with their distinguishing clinical features, are summarized in Table 5.

**TABLE 5:** Genetic disorders mimicking hypoxic–ischemic encephalopathy: clinical clues for differential diagnosis/genetic conditions associated with HIE phenocopies or increased susceptibility to hypoxic injury.

Gene	Mechanism	Clinical presentation	Distinguishing features	Key reference
*MECP2*	Epigenetic/synaptic dysregulation	Severe hypotonia, seizures < 24 h, apnea	Progressive microcephaly, absence of hypoxia markers	Liu et al. (19)
*CDKL5*	Synaptic dysfunction	Early seizures (<48 h), hypotonia	Refractory epilepsy, no sentinel hypoxic events	Fehr et al. (16)
*MTFMT*	Mitochondrial translation defect	Lactic acidosis, hypotonia	Normal cord gases, ineffective hypothermia	Pronicka et al. (21)
*KIF1A*	Axonal transport defect	Hypotonia, myoclonic seizures	Progressive cortical atrophy, degenerative imaging	Parobek et al. (4)

## A practical diagnostic algorithm for neonatal encephalopathy

NE requires a structured and time-sensitive diagnostic approach aimed at distinguishing HIE from alternative etiologies, particularly genetic and metabolic disorders. Early identification of non-hypoxic causes is essential to initiate targeted therapies and avoid inappropriate management, including unnecessary or ineffective therapeutic hypothermia. Importantly, early genetic and metabolic investigations should not delay the initiation of therapeutic hypothermia in neonates who meet standard eligibility criteria. Rather, these investigations should be initiated in parallel during the therapeutic window, particularly in cases with atypical clinical, biochemical, or neuroradiological features.

A stepwise diagnostic framework is proposed to guide clinicians in the early evaluation of neonates presenting with encephalopathy.

## Step 1: perinatal history and sentinel events

The initial assessment should focus on identifying evidence of perinatal hypoxic–ischemic insult. Key elements include sentinel events (e.g., placental abruption, uterine rupture), abnormal fetal heart rate tracings, low Apgar scores, need for prolonged resuscitation, and severe metabolic acidosis at birth. The absence of these features should raise suspicion for alternative etiologies. In addition, antenatal or fetal features such as fetal seizures (31), persistent fetal hiccups (32), reduced fetal movements (33), polyhydramnios (34), abnormal fetal tone (35), or abnormal antenatal MRI findings (36) may suggest non-hypoxic etiologies and should prompt early consideration of genetic or metabolic causes.

## Step 2: early clinical examination and red flags

A detailed neurological examination should be performed within the first hours of life. Certain clinical features may suggest a non-HIE etiology, including:

1. Onset of seizures within the first hours without clear perinatal distress
2. Severe hypotonia or abnormal tone not evolving according to typical HIE patterns
3. Dysmorphic features or congenital anomalies
4. Persistent apnea or autonomic instability
5. Family history of neonatal deaths or neurological disorders

## Step 3: neurophysiological assessment (EEG/aEEG)

Continuous EEG or aEEG should be initiated as early as possible. Specific patterns may provide diagnostic clues:

1. Burst-suppression patterns suggest genetic epileptic encephalopathies (e.g., *KCNQ2, STXBP1*)
2. Persistent severe background suppression without evolution may indicate metabolic or mitochondrial disorders
3. Electroclinical dissociation or unusual seizure semiology may support a genetic etiology

However, it should be noted that several EEG/aEEG patterns described in genetic or metabolic encephalopathies, including burst-suppression and severe background suppression, may significantly overlap with findings observed in severe hypoxic–ischemic encephalopathy and should therefore not be considered pathognomonic. Clinical correlation remains essential.

## Step 4: neuroimaging

Brain MRI, ideally performed within the first week of life, is crucial for distinguishing HIE from other conditions. Typical HIE patterns include basal ganglia–thalamic or watershed injury. In contrast:

1. Normal MRI or atypical findings should raise suspicion for genetic encephalopathies
2. Diffuse cortical malformations suggest disorders of neuronal development
3. Selective involvement of deep gray nuclei or brainstem may indicate metabolic or mitochondrial disease
4. Additional MRI/MRS findings that may suggest non-HIE etiologies include diffuse white matter abnormalities, isolated brainstem involvement, cerebellar abnormalities, or lactate peaks on magnetic resonance spectroscopy suggestive of mitochondrial dysfunction.

Several neuroimaging features associated with metabolic or genetic disorder, such as MR spectroscopy or diffuse signal abnormalities may also be observed in severe hypoxic–ischemic injury, further complicating interpretation.

## Step 5: first-line laboratory and metabolic work-up

Initial laboratory evaluation should include blood gas analysis, lactate, ammonia, glucose, electrolytes, liver function tests, and infection screening. Early metabolic testing is essential, particularly in the absence of clear hypoxic insult:

1. Hyperammonemia suggests urea cycle disorders
2. Lactic acidosis may indicate mitochondrial dysfunction
3. Abnormal amino acids or organic acids support inborn errors of metabolism

## Step 6: early genetic testing

In neonates without clear evidence of HIE or with atypical clinical, EEG, or MRI findings, early genetic testing should be considered. The initial “*expansion of screening*” refers to a tiered approach that begins with standard newborn screening (NBS) panels for common inborn errors of metabolism (e.g., amino acid disorders, organic acidaemias, fatty acid oxidation defects), followed by chromosomal microarray (CMA) to detect copy number variants, and targeted single-gene or small-panel testing when a specific diagnosis is strongly suspected on clinical grounds (e.g., pyridoxine trial with subsequent ALDH7A1/PNPO testing in drug-resistant seizures, or ammonia-guided OTC sequencing in suspected urea cycle disorders). When these first-tier investigations are non-diagnostic, broader genomic approaches are warranted. NGS, including targeted gene panels or WES, is increasingly used as a first-line diagnostic tool in selected cases. Rapid genomic testing—including ultra-rapid WES or WGS, now achievable within few days in specialized centers—may allow identification of treatable conditions such as vitamin-responsive epilepsies or metabolic disorders. The early integration of genomic testing should complement, rather than replace or delay, standard HIE management pathways.

## Step 7: therapeutic implications and re-evaluation

**FIGURE 1:**
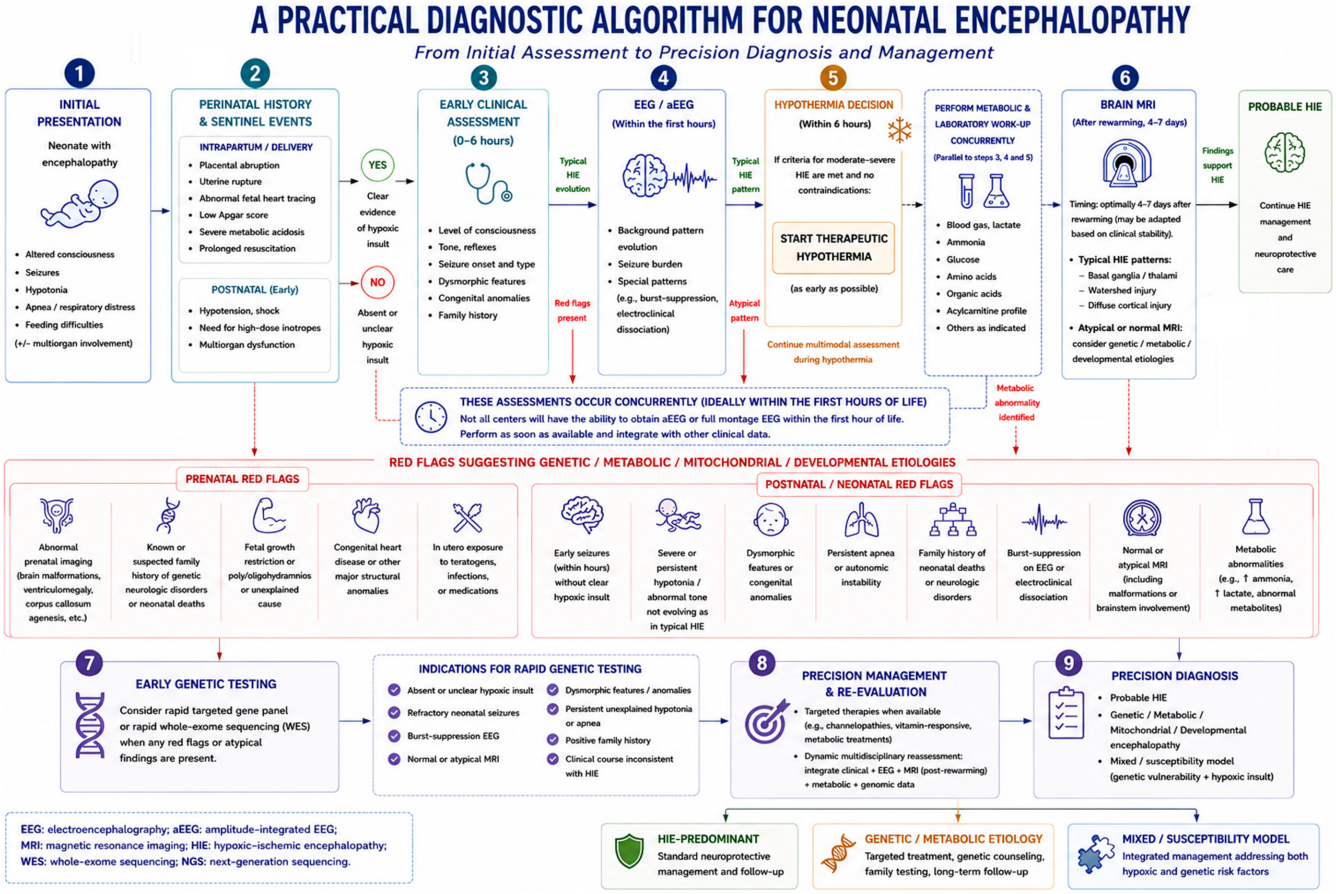
Operative flow-chart for Neonatal Encephalopathy.

The operative flow-chart for Neonatal Encephalopathy is reported in Figure 1.

## Therapeutic perspectives

Therapeutic hypothermia is effective only in a subset of patients with true HIE (3). In genetic encephalopathies, it may be ineffective or delay correct diagnosis.

Future strategies should focus on:

- genomic-guided therapies
- personalized medicine approaches
- early integration of genetic testing

Therapeutic hypothermia remains the standard of care for moderate to severe HIE, but its benefit is limited to neonates with confirmed hypoxic–ischemic injury. However, in neonatal practice, the decision to initiate therapeutic hypothermia is often made within the first hours of life, before genomic or metabolic results are available. In cases of genetic or metabolic encephalopathies, hypothermia may be ineffective and, in some instances, may delay the recognition of alternative diagnoses and the initiation of targeted treatments.. In this contest, given the substantial clinical overlap between hypoxic–ischemic and non-hypoxic encephalopathies, therapeutic hypothermia should not be delayed in eligible infants while further diagnostic investigations are ongoing. A parallel approach, combining timely initiation of cooling with early implementation of targeted diagnostic pathways, is therefore essential. The increasing recognition of genetic neonatal encephalopathies has significant therapeutic implications. Early identification of the underlying etiology enables the implementation of precision-based interventions that may substantially modify disease course and outcomes. Several genetic and metabolic conditions presenting with neonatal encephalopathy are potentially treatable, particularly when therapy is initiated promptly. These include vitamin-responsive epilepsies (e.g., pyridoxine- or pyridoxal phosphate-dependent seizures), thiamine transporter deficiencies, and urea cycle disorders, in which early metabolic correction can be life-saving. In epileptic channelopathies, treatment can be tailored according to the underlying molecular defect. For example, sodium channel blockers may be effective in specific sodium channel–related epilepsies, while other forms remain pharmaco-resistant and require alternative strategies. Similarly, mitochondrial and metabolic disorders may benefit from supportive metabolic therapies, cofactor supplementation, or dietary interventions, although outcomes remain variable. Despite these advances, significant real-world limitations must be acknowledged. Access to rapid genomic sequencing remains highly variable across healthcare systems and neonatal centers, with many resource-limited settings lacking the infrastructure for timely WES or WGS. Even in well-resourced environments, turnaround times for standard exome sequencing may extend to several weeks, limiting clinical impact during the critical acute neonatal period; ultra-rapid tests are not yet universally available. The cost of sequencing, the need for specialist bioinformatic support, and the challenge of interpreting variants of uncertain significance further constrain the routine applicability of precision genomic diagnostics. These barriers underscore the continued importance of first-tier clinical and metabolic investigations and highlight the need for equitable access frameworks and international collaboration in genomic medicine.

The rapid evolution of genomic technologies is reshaping the therapeutic landscape. The implementation of NGS, including rapid whole-exome sequencing, in the neonatal intensive care setting allows for earlier diagnosis and facilitates timely initiation of targeted therapies. Despite the increasing availability of rapid genomic technologies, limitations such as time to results, accessibility, and resource variability remain relevant in acute neonatal settings and should be considered within time-critical decision-making frameworks, including the 6-h window for therapeutic hypothermia in moderate-to-severe encephalopathy. Genetic investigations should therefore be integrated into a parallel diagnostic approach without delaying established first-line interventions. In parallel, emerging approaches such as gene therapy, antisense oligonucleotides, and RNA-based treatments are being investigated for selected monogenic disorders, although most remain at a preclinical or early clinical stage. Future strategies should therefore focus on the integration of genomic data into clinical decision-making, the development of disease-specific therapies, and the establishment of standardized diagnostic pathways that incorporate early genetic testing. A precision medicine approach, combining clinical, neurophysiological, metabolic, and molecular data, is essential to optimize treatment, avoid ineffective interventions, and improve both short- and long-term neurological outcomes in neonates with encephalopathy.

## Conclusion

Neonatal encephalopathies require a major paradigm shift. Not all cases are hypoxic–ischemic, and many have a genetic origin, some of which are treatable if identified early. The integration of genomic sequencing with clinical, neurophysiological, and metabolic data enables a precision medicine approach, improving diagnosis, treatment, and long-term outcomes. At the same time, this approach must be integrated within time-critical clinical decision-making frameworks, ensuring that established therapies such as therapeutic hypothermia are not delayed while advanced diagnostics are pursued. Emerging evidence suggests that genetic background may influence both susceptibility and response to hypoxic–ischemic injury. Variants affecting mitochondrial function, oxidative stress pathways, excitotoxicity, or neuroinflammatory regulation may lower the threshold for neuronal injury and contribute to interindividual variability in clinical severity and neurodevelopmental outcomes following comparable hypoxic insults. The increasing availability of genomic sequencing, combined with the integration of neuroimaging, EEG, metabolic, and inflammatory data, now enables a multidimensional diagnostic approach oriented toward precision medicine. This approach has a direct impact not only on prognosis and treatment, but also on the quality of family counseling, the avoidance of ineffective therapies, and access to targeted care. In the coming years, it will be crucial to implement clinical algorithms in neonatal practice that integrate early sequencing, molecular modeling, and individualized therapeutic targeting. Because neonatal genetic encephalopathies may overlap clinically and mechanistically, the proposed classification is intended as a practical functional framework based on predominant pathophysiological and clinical features rather than mutually exclusive categories
